# A Rare Form of Heteropagus Twinning: Three-Armed Infant with Spinal Dysraphism

**DOI:** 10.1155/2012/831649

**Published:** 2012-12-12

**Authors:** Aynur Solak, Sonnaz Ergün, İpek Polat, Neslın Şahin, Berhan Genç

**Affiliations:** ^1^Radiology Department, Sifa University, Izmir, Turkey; ^2^Department of Pediatrics, Sifa University, Izmir, Turkey; ^3^Department of Pediatrics, Dokuz Eylul University, Izmir, Turkey

## Abstract

An ectopic or accessory limb attached to the back is an extremely rare and strange condition, and there are only a few documented cases in the worldwide literature. The first case was described by Jones and Larkin (1889). There are several theories regarding the origin of this condition. Asymmetric conjoined twinning which is located dorsally in the vertebral column (rachipagus) is the most probable diagnosis of our patient. Conjoined twinning is very rare and the incidence is 1 per 50 000 live births. Rachipagus is even rarer, with no more than 30 case reports so far (Chadha et al. (1993, 2006)). In this report, we present a patient who underwent successful surgical excision of a third arm attached to the back in the midline over the low-dorsal region. Differential diagnoses including teratoma and fetus in fetu are discussed.

## 1. Case Report

A female baby was born by C/S after an uncomplicated full-term pregnancy, weighing 3500 g with Apgar scores 8 and 9 at the first and fifth minutes, respectively. The patient did well; she was referred to our Pediatric Care Unit.

Antenatal course was uneventful and no history of maternal medication or harmful drug use or exposure to radiation during pregnancy was reported. Her mother was followed up during pregnancy. She had several prenatal ultrasound examinations, but her daughter's abnormality was diagnosed postnatally.

**Figure 1:**
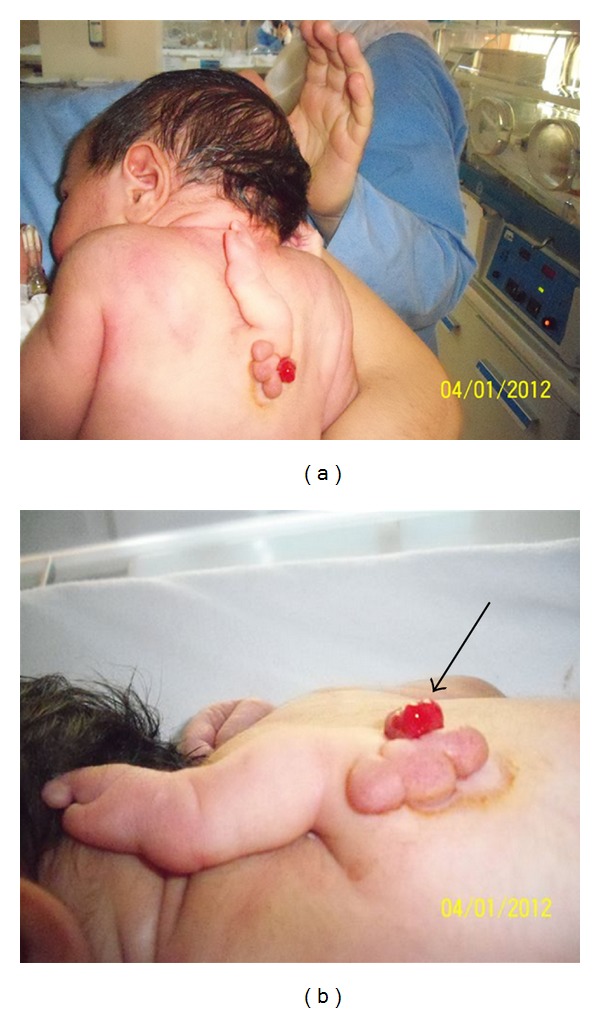
A malformed accessory arm attached to the back; it is immobile. There are three small buds originating from just below the root of the arm (a). Note that hemorrhagic area is located caudal to this accessory extremity (arrow).

On physical examination, a malformed accessory extremity, resembling a rudimentary structure like a tiny arm, was attached to the back, in the upper-dorsal region of the vertebral column (Figures 1(a) and 1(b)). The accessory limb could not be moved. There was no arterial pulse in this ectopic limb. The baby had no neurological deficit or evidence of any other congenital abnormality. 

**Figure 2:**
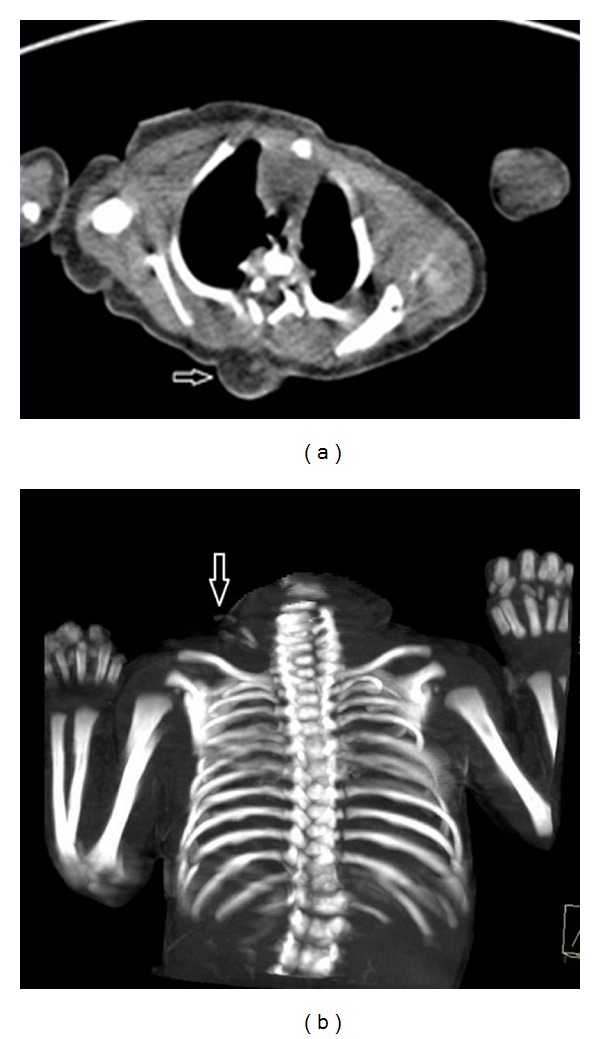
Axial tomographic section (a) shows spina bifida adjacent to the root of the ectopic extremity (arrow). Coronal reformatted image (b) shows two hypoplastic metacarpals and three phalangeal bones of the hand (arrow).

Abdominal and cranial ultrasonography (USG), thoracic computed tomography, and magnetic resonance imaging (MRI) were performed. Cranial USG showed no abnormality. Brain and medulla spinalis were normal in MRI except a 2 cm lipoma located at the same level with the origin of the ectopic extremity. There was no associated meningocele or myelocele. Abdominal organs appeared normal on USG. Thoracic CT revealed spina bifida from 5th cervical to 7th dorsal vertebrae level (Figure 2(a)). Coronally reformatted tomographic images revealed that the limb had two hypoplastic metacarpal and three phalangeal bones with no humerus, radius, or ulna (Figure 2(b)). There were no genetic abnormalities in pedigree analysis.

**Figure 3:**
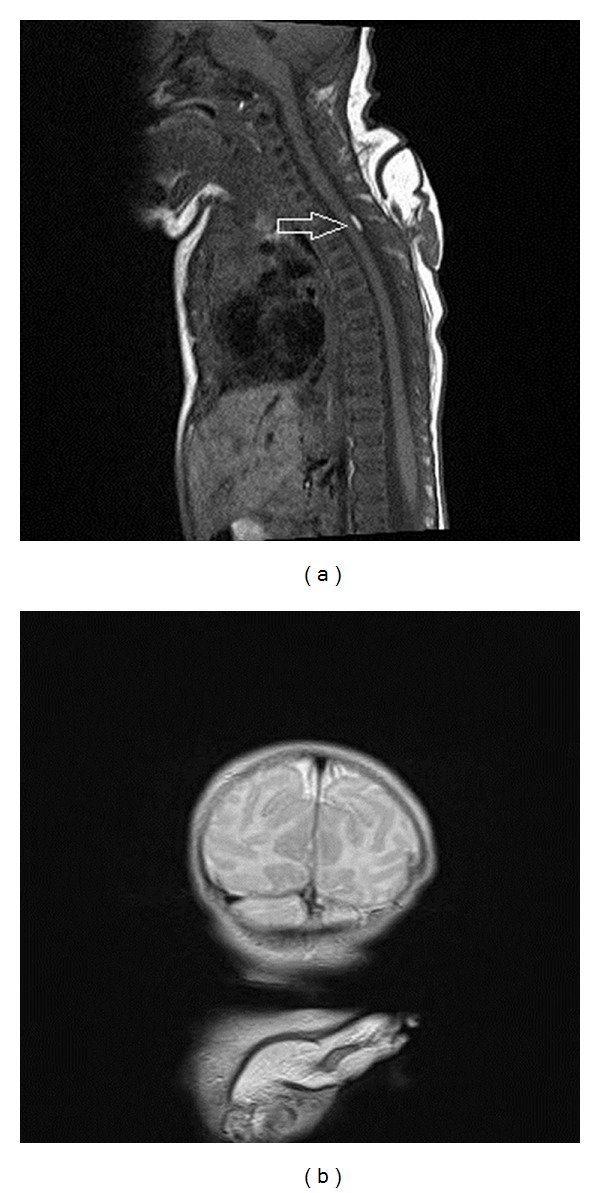
T1-weighted sagittal (a) and T2-weighted coronal (b) images. Arrow points that the lipoma is located at the same level of the ectopic extremity. Medulla Spinalis terminates between L3 and L4 vertebrae. Ectopic extremity originates from subcutaneous soft tissues and it does not extend deeper neural elements. It ends interspinous ligament. Coronal T2-weighted image shows dorsally located extremity, composed of fat and muscle tissue. Note that cerebral structures are in normal limits.

The MRI demonstrated that medulla spinalis terminated between L3 and L4 vertebraes. Ectopic arm was originated from subcutaneous soft tissues and it did not extend to deeper neural elements. It ended with interspinous ligament (Figures 3(a) and 3(b)). Routine biochemical blood tests and urine analysis were normal. Echocardiography did not reveal any abnormality.

Surgical operation was performed when the baby was two months old. Midline skin incision was performed between the first and the seventh thoracic vertebrae of the dorsal region. Incision extended around the accessory extremity so that the periphery of the mass and its globular appendages were liberated. The mass was attached firmly to the laminae of the dorsal vertebrae. It was excised totally. There was no relationship between the mass and the underlying dura mater, and intraspinal structures. A significant closure defect was observed at the level of the fifth thoracic vertebrae, and a single level laminectomy was made using a Kerrison punch. Dura mater was incised and a spinal lipoma was observed. However, it was wrapped tightly around the nerve fibers; thus it could not be excised. Finally, closure of the subcutaneous tissues and skin was performed. The infant did not require postoperative ventilation and made an uneventful recovery. She began oral feeding with no complications on the same day, six hours after the operation. She was discharged on the fourth day of hospitalization. Histopathological examination revealed normal muscle tissue extending along the ectopic extremity and three bones resembling metacarpal bones and phalanges. There were no neural, cartilaginous, or vascular tissues. At the bottom of the mass, the lesion was covered by a thin-walled hemorrhagic mucosa. Microscopically, a primitive type intestinal mucosa resembling colonic mucosa was noted in this region. DNA analysis was performed. DNA samples from the blood and hair of the autosite and from the skin and muscle of the parasitic twin were extracted using the rapid Chelex-100 method. A sex locus (amelogenin gene) and 15 STR loci were amplified using the Powerplex TM 16 system. DNA analysis revealed that the parasitic twin in this report had an identical genotype to the autosite, which indicated that it was of monozygotic origin. The patient is currently 14 months old and completely healthy.

## 2. Discussion

Heteropagus is a term used to describe the development of an asymmetrical form of twinning when one of the twins monopolizes the placental blood at the expense of other fetus with consequent ischaemic atrophy of the latter [1]. Some parts of the damaged fetus are attached to the partner's body, continuing to grow and developing like a parasitic organism [1, 2]. Conjoined twinnings are classified in terms of the attachment site of the body: thoracopagus (thorax), ischiopagus (pelvis), cephalopagus (face), omphalopagus (abdomen), and craniopagus (cranium) [3, 4]. Rachifagus describes a parasitic twin joined dorsally at the vertebral column. If the conjoined region is located at the lumbosacral spine, it is called pygopagus. Sometimes a parasitic fetus may completely develop and connect to partner's body via laminae of the thoracic vertebrae. Sometimes, depending on the severity of the pathology, only a mass of viable tissue may remain [4–6]. The most widely accepted theory explaining the embryogenesis of this abnormal condition is the fusion hypothesis. In the early fetal life, embryonic discs of monozygotic monoamniotic twins are located in the same amniotic cavity. At the third or fourth gestational weeks, the neural folds of the two different embryos can merge if the skin covering the neural tube gets damaged. If the two embryos develop fair and equally, two complete but conjoined fetuses arise [7]. But, in most cases, one of the twins dies and some parts of its body can remain attached to the vertebral column of the other fetus [1, 7]. This attached part composed of primitive embryonic tissue prevents closure of the neural tube during later development, resulting in spina bifida, or other neural tube defects [4, 8]. Thus, the most anticipated anomaly of the living fetus is the closure defect of the dorsal vertebral column. Location of the spinal lipoma at the same level and the origin of the limb support this explanation. Important abnormalities involving other organ systems are rare in the living fetus. In patients with rachipagus, a lipoma at the base of the parasitic mass with an intraspinal extension has frequently noticed [1, 2, 4, 7]. In the case presented here, the mass was firmly attached to the posterior elements of the fourth and fifth vertebrae, and the intraspinal lipoma was found at the same level.

Based on the typical appearances and histological results, diagnosis of rachipagus is made easily but rarely the differential diagosis may be required from teratoma and fetus in fetu [9, 10]. Teratoma is defined as a true tumor arising from the uncontrolled growth of pluripotent stem cells [8, 11]. Sometimes, teratomas may contain mature tissues such as respiratory epithelium, fat tissue, hair follicles, fingers, teeth, and jawbones. However, these bones are scattered throughout the tumor and never resemble the real limb. If the fetus is located inside the body of its twin, this process occurs in the other twin's body cavities; this condition is termed fetus in fetu [10–12]. Sometimes, a malformed, abnormal fetus enveloped inside its twin may remain totally silent until later ages, or it can cause an intra-abdominal or sometimes an intrathoracic mass. However there have been a few reports of parasitic twin location in the head, sacrum, scrotum, and even in the oral cavity. Presence of vertebral axis and appropriate arrangement of other organs or limbs in its relation are the criteria to distinguish a fetus in fetu from a rachifagus and highly differentiated teratoma [13–16]. We did not find any vertebral structures in our patient's specimen. The appearances, the external-midline location of the accessory limb, and the presence of colonic mucosal cells suggested the presence of conjoined twinning. However, teratomas occurring in association with a fetus in fetu and rachiphagus have been reported in the literature. Thus, such a clear distinction of these entities may not be true at all times [11, 17].

Neural tube defects associated with ectopic limbs have been reported in a few cases. Krishna et al. reported accessory legs associated with spina bifida and rudimentary external genitalia [18]. Sharma et al. reported two of three cases with spina bifida and hemivertebrae [19]. Zhang et al. reported rachiphagus conjoined twinning in a seventeen-year-old female with spina bifida, tethered cord, diplomyelia, scoliosis, and ventricular septal defect. They observed a well-developed breast attached to the back in the midline at low-thoracic region [7]. We did not find any other osseous anomaly in the vertebral column. Unlike teratomas, 6%–10% of which are malignant, rachipagus is regarded as a benign condition [8, 10]. Treatment is surgical. To date, no case of both twins living has been reported. Thus, parasitic twin excision is sufficient for treatment, and it does not require complicated separation procedures [1, 7]. 

In summary, a case of rachifagus is presented herein, which had the limb buds very similar to extremities, was located at the midline of the dorsal region, and attached to the vertebral column. The literature of this amazing condition was reviewed. We concluded that this anomaly was limited to the infant's dorsum. Nevertheless, other midline pathologies including vertebral column, medulla spinalis, and heart should be kept in mind. Thus, careful preoperative examination of the patient is essential for a good postoperative outcome.
